# Sulphurous air pollutants and exposure events of workers in thermal-mineral springs: a case study of *Contursi Terme* (Salerno, Italy)

**DOI:** 10.1007/s11356-022-22432-y

**Published:** 2022-08-09

**Authors:** Concetta Pironti, Maria Ricciardi, Oriana Motta, Marta Venier, Antonio Faggiano, Raffaele Cucciniello, Antonio Proto

**Affiliations:** 1 Department of Medicine Surgery and Dentistry, University of Salerno, via S. Allende, 84081 Baronissi, SA Italy; 2 O’Neill School of Public and Environmental Affairs, Indiana University, Bloomington, IN USA; 3 Department of Chemistry and Biology, University of Salerno, via Giovanni Paolo II 132, 84084 Fisciano, SA Italy

**Keywords:** Occupational health, Pollutant exposure, Air monitoring, Thermal-mineral springs

## Abstract

Thermo-mineral springs are widely spread over the volcanic areas of Salerno, a city in southern Italy. Although the water of thermal structures provides beneficial effects on human health, the air is characterized by the presence of potentially toxic compounds, such as hydrogen sulphide (H_2_S) and sulphur dioxide (SO_2_). Exposure to sulphurous compounds may have detrimental effects on human health, with asthma being the most common. In this study, air concentrations of H_2_S and SO_2_ in the thermal springs of *Contursi Terme* (Salerno, Italy) were monitored for 4 months (using both active and passive sampling), along with the chemical and microclimatic characterization of thermal water, to assess workers’ exposure to these pollutants. An in-depth characterization of indoor air at the springs is paramount to establish emission control limits for occupational exposure and to take protective measures. The air concentration of SO_2_ varied from 0.11 ± 0.02 to 0.91 ± 0.02 mg/m^3^, following a seasonal pattern (higher values in winter and lower in spring). Conversely, indoor H_2_S concentrations did not vary significantly with time, but outdoor levels (from 0.40 ± 0.03 to 1.90 ± 0.03 mg/m^3^) were always higher than indoor ones (from 0.11 ± 0.03 to 0.56 ± 0.03 mg/m^3^). Not negligible air concentrations of these pollutants were detected in this thermal spring workplace, so further investigations are needed to ensure workers’ safety.

**Supplementary Information:**

The online version contains supplementary material available at 10.1007/s11356-022-22432-y.

## Introduction

Thermal springs are mineral waters with a specific saline composition (Quattrini et al. 2016). These environments have been known as early as the first century A.D. when their presence was reported in ancient literature and the use of thermal baths for curative purposes was well known since Roman times (Routh et al. 1996; Frosch 2007; Valeriani et al. 2018). The thermo-mineral springs received great attention in the culture of bathing and personal hygiene for specific therapeutic uses (Jackson 1990; Croutier 1992; Frost 2004; Torres-Ceron et al. 2019; Costantino et al. 2020). Although the water of thermal structures provides beneficial effects on human health, the atmosphere of these environments is characterized by the presence of sulphurous compounds such as hydrogen sulphide (H_2_S) and sulphur dioxide (SO_2_) (Attene-Ramos et al. 2006; Stanhope et al. 2017; Pironti et al. 2021a, b ; Ricciardi et al. 2021). H_2_S is an undesirable air pollutant because of its malodour and toxicity even at low concentrations (< 10 ppm) (Elwood 2021). Concerns about health effects are mostly related to the brain and central nervous system, with the risk of damage depending on both the length of the exposure and the concentration of H_2_S (EPA 1986; Legator and Singleton 1997; Lim et al. 2016; Nuvolone et al. 2019).

Air pollutants could interact with the body via skin contact, the respiratory tract, or even oral intake, which pose various health hazards, such as respiratory irritation (Rafieepour et al. 2013) and oral disease (Vianna et al. 2005), and may even increase the risk of cancer (IARC 1992; NTP 2016; Ghantous et al. 2015). Irritants such as acid gases are less known than sensitizers as causative agents of occupational respiratory diseases, which hampers the recognition and the understanding of the hazards of irritative agents at workplaces. The health impact of irritants is potentially high because persistent symptoms and abnormal lung function have still been reported years after diagnosis. Recently, the role of irritants in pulmonary disease has also been discussed (Dumas et al. 2014). Many studies on occupational respiratory hazards related to the presence of irritants in different industries’ workplaces (gases, dust, fumes, mists, vapours, smoke, fog and sprays) were reported in the literature. In some cases, substances are generated via industrial processes, for example during the aeration process, drying of the sludge and mechanical filtering processes.

SO_2_ is another important air pollutant to monitor in workplaces for worker safety (Goudarzi et al. 2016; Yan et al. 2020; Wang et al. 2022). It is produced from the combustion of solid fossil fuels and is considered the most relevant pollutant from materials’ deterioration, especially in the corrosion of metals and stone recession. These corrosive effects are even greater with the presence of an oxidizer such as NO_2_. SO_2_ is the most important pollutant from industrial activities such as petroleum refining, non-ferrous metal smelting and burning of coal for energy production. Exposure to SO_2_ can result in an increased risk of lung cancer (Lee et al. 2002; Hamra et al. 2015) and heart and respiratory diseases (Golbaz and Jonidi Jafari 2011; Shang et al. 2013; Beelen et al. 2014; Dursun et al. 2015).

In recent years, despite a general improvement in air quality in workplaces, a real concern over the preservation of the health of workers exposed to atmospheric pollution remains (Charlier et al. 2021; Motta et al. 2021; Motta et al. 2022a; Montano et al. 2022; Nascimento et al. 2020; Zhang et al. 2021). Even though several studies regarding the beneficial effects of thermal waters for users are recognized in the literature (Giampaoli et al. 2013; Carbajo and Maraver 2017; Costantino et al. 2020), only one study so far has monitored the air concentration of pollutants such as H_2_S in thermal spring environments (Fazlzadeh et al. 2018), while no studies have investigated SO_2_ concentration in the air of this specific workplace. Obviously, the understanding of the origin and the evolution of contaminants is necessary for the decisions that must be taken by industrial companies and international agencies of health (Mohai et al. 2011; Vimercati et al. 2018; Motta et al. 2022b; Pironti et al. 2022).

In this study, we monitored the concentration of specific pollutants (H_2_S and SO_2_) at the thermal springs of *Contursi Terme* (Salerno, Italy) for 4 months to evaluate workers’ exposure to these harmful pollutants and evaluate the need to implement corrective measures to safeguard workers’ health.

## Material and method

### Materials

All the reagents used for the measurements (Na_2_CO_3_, NaHCO_3_, H_2_SO_4_, Na_2_SO_4_, H_2_O_2_, NaOH, thiosulphate, iodine, certified reference material, silica gel) were purchased from Sigma-Aldrich (St. Louis, MO, USA).

### Sampling site

**Fig. 1:**
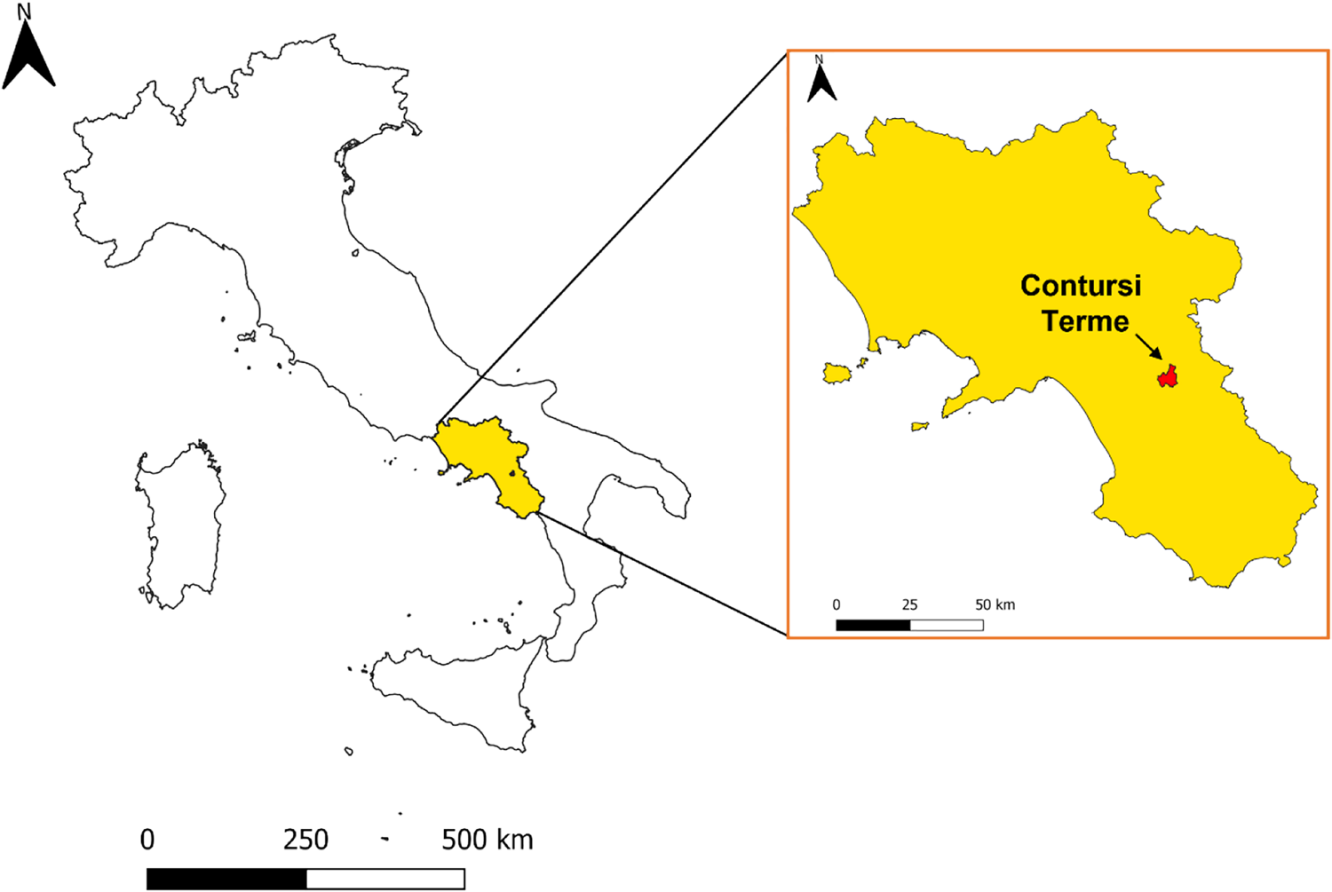
Map of Italy: magnification of the Region Campania is shown in yellow, while the village of *Contursi Terme* is shown in red

Water and air sampling was performed from 21 January to 21 April 2015 in the thermal springs of *Contursi Terme* (Salerno, Italy). The location of this village in the Region Campania of Italy is shown in Fig. 1.

With a temperature at the source of 47.6 ± 0.5 °C, the thermal spring water at Contursi Terme can be classified as hyperthermal (Štambuk-Giljanović 2008; Quattrini et al. 2016). Monthly water samples were taken from the pool of the thermal springs during the sampling period. Physicochemical characteristics of the thermal water were monitored by measuring the concentration of cations (Li^+^, Na^+^, K^+^, Ca^2+^ and Mg^2+^), anions (Cl^−^, SO_4_^2−^ and HCO_3_^−^), silica and sulphide; conductivity; fixed residue at 180 °C; hardness; temperature; and pH at the source and pool. The temperature, conductivity and pH of water samples were determined using a multiparameter probe from Hanna Instruments (HI98194).

### Passive and active air monitoring

Air monitoring was conducted using both active and passive samplers. Passive samplers employed were RING® radial diffusive devices (purchased from Aquaria Srl, Milan, Italy) (Cucciniello et al. 2012, 2015; Proto et al. 2014; Motta et al. 2018). Bar and restaurant rooms were considered indoor environments while the external pools as outdoor environments. H_2_S and SO_2_ were measured according to the methodologies of the National Institute for Occupational Safety and Health (NIOSH), the US federal agency for research and prevention of work-related injury and illness (Methodology 6013 for Hydrogen Sulphide and 6004 for Sulphur Dioxide) (NIOSH 1994a, b).

Active sampling was carried out near the thermal spring source (1 m away from the thermal source) with a sorbent tube and an AP Buck VSS 1 pump (Aquaria srl, Italy) using an airflow rate of 200 mL/min (Cucciniello et al. 2017). Active monitoring was done two times per month for exposure times of 20–30 min. Passive sampling was performed in the bar and restaurant room (indoors), and external pool (outdoors). For each passive sampler, the start and the end of sampling period coincide with the days in which active sampling was performed, so the exposure time is in the range 7–14 days.

A detailed description of all the collected samples, including sampling date, exposure time, weather conditions and number of samples, is reported in Table S1. For each sample type (SO_2_ active sampling, H_2_S active sampling, SO_2_ passive sampling indoor, SO_2_ passive sampling outdoor, H_2_S passive sampling indoor and H_2_S passive sampling outdoor), samples are enumerated in chronological order from January to April.

The substrate for the H_2_S monitoring in the air was based on zinc acetate–impregnated silica in glass tubes. After collection, H_2_S is oxidated to sulphate with an alkaline solution of hydrogen peroxide according to previous work (Motta et al. 2014). Triethanolamine was used as the substrate for SO_2_, and then water extraction was performed and SO_2_ was recovered as sulphate.

### Chromatographic analyses

Target chemicals (SO_2_ and H_2_S) were quantified as sulphate using ion-exchange chromatography on a Thermo Scientific Dionex™ Aquion™ ion chromatograph equipped with a conductivity system detector (Ricciardi et al. 2022). A Dionex IonPac AS23 carbonate eluent anion-exchange column was used for anions (Cl^−^ and SO_4_^2−^), while a Dionex IonPac CS12A (sulphuric acid as eluent) for cations (Li^+^, Na^+^, K^+^, Ca^2+^ and Mg^2+^). Ionic concentrations (expressed as mg/L) were obtained using calibration curves prepared employing standard ion solutions. The precision, expressed as one standard deviation, was 1% for all the ions considered.

### Sulphidimetric grade determination

Evaluation of total and dissolved sulphide in water was done according to national protocol APAT IRSA-CNR (APAT IRSA-CNR 2003):

$$\mathrm S^{2-}\left(\frac{\mathrm{mg}}{\mathrm L}\right)=\frac{\left({\mathrm{aN}}_{\mathrm I}-{\mathrm{bN}}_{\mathrm T}\right)16\times1000}{\mathrm V}$$

where *a* is the volume (mL) of the iodine solution used in the titration, *b* is the volume (mL) of the thiosulphate solution used in the titration, *N*_I_ is the normality of the iodine solution, *N*_T_ is the normality of the thiosulphate solution, *V* is the volume (mL) of the sample taken, and 16 is the equivalent weight of sulphide.

For the determination of the dissolved sulphide content, a preliminary separation of the suspended sulphides by sedimentation was carried out, making them flocculate by the addition of a solution of aluminium chloride and sodium hydroxide.

### Statistical analysis

Statistical analyses, including one-way ANOVA (analysis of variance), were performed using the R Studio software (version 4.1.1). In particular, we evaluated the statistical differences between the indoor and outdoor concentrations of the considered pollutants obtained by passive sampling and the statistical differences between the concentrations recorded in different sampling periods. The null hypotheses for the ANOVA were that there are no differences between indoor and outdoor concentrations detected for the same pollutant during the same sampling period and there are no differences between concentrations recorded in different sampling periods. Hence, the independent variables were the “type of environment” (indoor and outdoor) and the “sampling period” (January–February and March–April), whereas the dependent variable was the air concentration of the considered pollutants. The significance level was set at *α* = 0.05.

## Results

The results of the physicochemical analysis of water are summarized in Table 1. The bicarbonate sulphurous mineral thermal water presented concentrations of HCO_3_^−^ equal to 1800 ± 40 mg/L, SO_4_^2−^ of 270 ± 10 mg/L and a total sulphide content of 28 ± 2 mg/L (dissolved sulphide was 16 ± 1 mg/L).

**Table 1:** Physicochemical characteristics of the thermal springs; averages with the standard deviation (*σ*) are reported

Parameter		Result
Cations (mg/L)	Li^+^	3 ± 1
	Na^+^	730 ± 30
	K^+^	150 ± 10
	Ca^2+^	340 ± 20
	Mg^2+^	140 ± 10
Anions (mg/L)	Cl^−^	990 ± 30
	SO_4_^2−^	270 ± 10
	HCO_3_^−^	1800 ± 40
Silica (mg/L)	SiO_2_	38 ± 5
Total sulphide (mg/L)	S^2−^	28 ± 2
Dissolved sulphide (mg/L)	S^2−^	16 ± 1
Conductivity at 20 °C (µS/cm)		5620 ± 60
Fixed residue at 180 °C (mg/L)		3870 ± 70
Water hardness (°F)		137 ± 8
Temperature at source (°C)		47.6 ± 0.5
Temperature at pool (°C)*		27 ± 1
pH at source		6.67 ± 0.05
pH at pool*		6.9 ± 0.1

^*^Values measured in January

The atmospheric pollutants (SO_2_ and H_2_S) were measured at different times and locations in the thermal spring sites. First, active samples were collected to measure the concentration of pollutants in a short time interval (20–30 min) and in a specific place. Air concentrations of SO_2_ and H_2_S obtained by active sampling were in the range 2.0–5.2 mg/m^3^ and 2.2–20.2 mg/m^3^, respectively (Table 2). These values are snapshots of the pollutant’s concentrations near its emission source.

**Table 2:** Air concentration values of SO_2_ and H_2_S (mean of three values) obtained by active sampling, with the standard deviation (*σ*)

Sample	Analyte	Air concentration (mg/m^3^)	*σ*
1	SO_2_	4.9	± 0.3
2	SO_2_	3.0	± 0.2
3	SO_2_	5.1	± 0.3
4	SO_2_	3.9	± 0.3
5	SO_2_	4.1	± 0.3
6	SO_2_	2.0	± 0.3
7	SO_2_	2.2	± 0.4
8	SO_2_	5.2	± 0.3
9	H_2_S	19.0	± 0.3
10	H_2_S	16.9	± 0.4
11	H_2_S	20.2	± 0.4
12	H_2_S	6.1	± 0.3
13	H_2_S	8.9	± 0.4
14	H_2_S	2.2	± 0.3

To evaluate the mean concentration of a pollutant to which workers are exposed daily and temporal variations, passive sampling was performed both indoors (bar and restaurant room) and outdoors (external pool). The use of passive samplers allows to obtain threshold limit value-time-weighted averages (TLV-TWA) of the concentrations of pollutants in a wider time lapse than active sampler (168–336 h vs 20–30 min) and to acquire average concentrations over time. In fact, contrary to active sampling, the values resulting from passive sampling are not susceptible to punctual emissions and momentary variations of pollutant concentration.

**Fig. 2:**
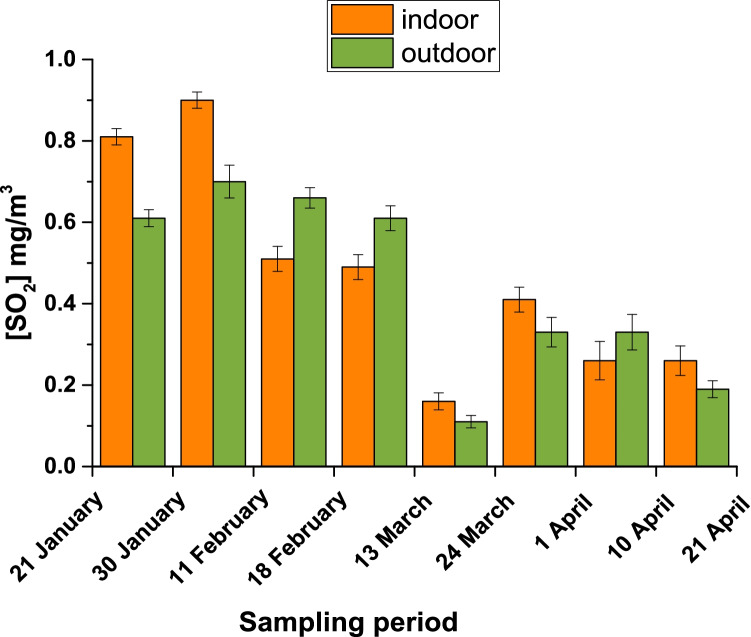
SO_2_ values in indoor (yellow) and outdoor (green) environments by passive sampling

**Fig. 3:**
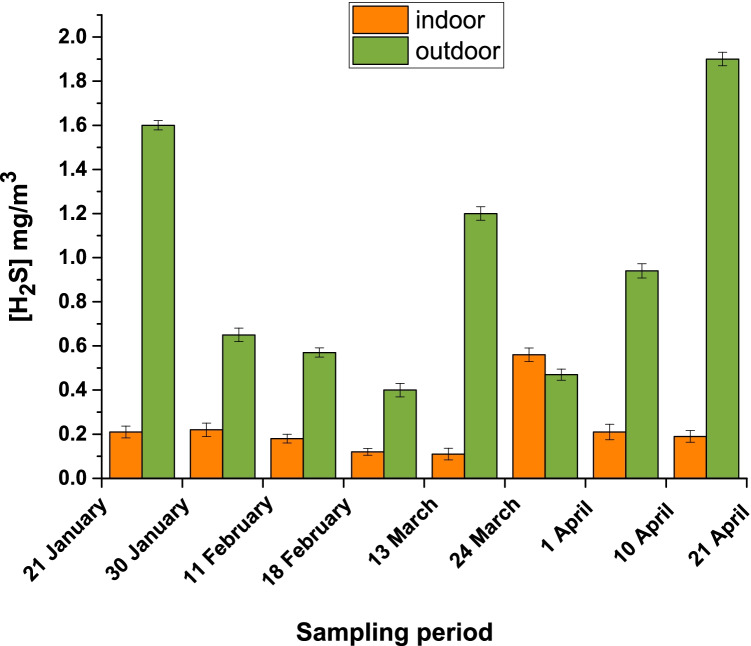
H_2_S values in indoor (yellow) and outdoor (green) environments by passive sampling

The temporal variation in concentrations measured by passive sampling during the monitoring campaigns is shown in Fig. 2 for SO_2_ and in Fig. 3 for H_2_S for indoor (yellow) and outdoor (green) environments. SO_2_ and H_2_S concentrations detected by passive sampling are generally lower than those obtained by active sampling and varied from 0.11 to 0.91 mg/m^3^ and from 0.11 to 1.90 mg/m^3^, respectively.

The ANOVA showed there are no significant differences (*p-value* > 0.05) between the indoor and outdoor concentration values for SO_2_, whereas there are considerable differences (*p-value* < 0.05) between the indoor and outdoor concentrations for H_2_S. Moreover, there are significant differences between the concentrations detected during the first half of the sampling period—January to February—and those recorded in the second half—March to April—for SO_2_ (*p-value* < 0.05), but not for H_2_S (*p-value* > 0.05).

## Discussion

Because the effects of pollutant exposure on human health can become visible only after several years, when the action measures are necessary but also difficult to take, a prevention strategy is crucial to protect human health in workplaces, where people spend most of their daily time. The importance of this study is linked to the determination at this thermal site of the presence and levels of certain types of pollutants that, even at low concentrations, can have lasting harmful effects on human health over time.

In this study, we looked at two pollutants that are most representative of thermal water composition, H_2_S and SO_2_. To obtain a full overview of the concentration of pollutants at this thermal site, both active and passive air sampling were performed. For both pollutants, the concentrations detected by active sampling (Table 2) were higher than those recorded by passive sampling (Table S2 and Figs. 2 and 3). This is in line with the principle that active sampling gives a point measure of concentration and not a time average. Moreover, active sampling was carried out near the thermal spring source (at the entrances of the hot water into the pool), whereas passive samplers were placed in the most crowded places with customers and workers, near the pool and the workstations to better evaluate the concentration at which customers/patients and/or workers are exposed.

During the monitoring campaigns, we noted a decreasing trend in the concentration of SO_2_ (Fig. 2) from January to February (means of 0.65 ± 0.04 mg/m^3^ for outdoor and 0.7 ± 0.2 mg/m^3^ for indoor) to March–April (means of 0.2 ± 0.1 mg/m^3^ for outdoor and 0.3 ± 0.1 mg/m^3^ for indoor), without significant differences between the indoor and outdoor environments (*p-value* > 0.05). The presence of this pollutant is mainly related to anthropogenic emissions from industries and household heating, and not to the thermal spring itself.

The air concentration values for SO_2_ obtained by passive sampling were always lower than the exposure limits in workplaces, expressed as both TLV-TWA (threshold limit value-time-weighted average), that is 5.2 mg/m^3^, and TLV-STEL (threshold limit value-short-term exposure limit), that is 13.3 mg/m^3^. Conversely, active sampling showed air concentrations closer to the TLV-TWA limit. In literature, studies involving exercising asthmatics indicate that a proportion of the population experience changes in pulmonary function and respiratory symptoms after periods of exposure to SO_2_ as short as 10 min. For instance, in Canada an increase of 11% in hospitalizations due to respiratory diseases from 1995 to 2000 in children from 0 to 14 years was reported after exposure to 10 μg/m^3^ of sulphur dioxide (Li et al. 2019). Based on this evidence, the World Health Organization (WHO) air quality guidelines revised the SO_2_ guideline, adopting a prudent precautionary limit of 20 µg/m^3^ for 24-h periods and a value of 500 µg/m^3^ for 10-min averages (World Health Organization 2006). Moreover, industrial activities, e.g. oil and gas extraction, contribute to the increase of air concentration of H_2_S and SO_2_ in rural areas (Burstyn et al. 2007). In Italy, regulatory limits are set to 350 µg/m^3^ as the hourly average (not to be exceeded more than 24 times per calendar year) and 125 µg/m^3^ for daily averages (not to be exceeded more than 3 times per calendar year) (D.Lgs 155/2010 2010). All the recorded concentrations of SO_2_ in this study exceeded these national limits.

In these thermal-mineral springs, various effects are present at the same time: water, high humidity and temperature. These conditions promoted SO_2_ solubility in water to give an acidic solution, capable of reacting with other chemical compounds present in that environment. These representative pollutants are strongly linked to an increase in temperature and visitors in correspondence to spring. Moreover, the indoor environments (reception, bar, restaurant room etc.) were characterized by the presence in the air of acidic pollutants such as SO_2_ and H_2_S that cause corrosion of metals present in electronic devices, resulting in faster degradation of the indoor environments. Both SO_2_ and H_2_S are strongly corrosive agents, so their presence in indoor air must be monitored to prevent the degradation of metal-containing devices (Cox and Lyon 1994; Kobus 2000; Wen et al. 2018).

On the other hand, in the case of H_2_S (Fig. 3), no significant variation of concentrations was noticed between the sampling period of January–February and that of March–April (*p-value* > 0.05), while a significant difference between indoor (overall mean of 0.23 ± 0.05 mg/m^3^) and outdoor (overall mean of 1.0 ± 0.5 mg/m^3^) concentrations was observed (*p-value* < 0.05). This is representative of the fact that the presence of H_2_S is specific to the thermal springs.

European and national legislation do not define limit values or air quality target values for H_2_S. In the absence of specific regulatory references, it is standard practice to refer to the WHO guideline values. The atmospheric concentration limit values are 7 ppm (9.76 mg/m^3^) for a 30-min average to olfactory pollution and 150 ppm (209 mg/m^3^) for a daily average to prevent eye irritation (World Health Organization 2003). Furthermore, in Europe the Scientific Committee on Occupational Exposure Limits (SCOEL) recommended a TLV-TWA of 5 ppm (7 mg/m^3^) and a TLV-STEL of 10 ppm (14 mg/m^3^) (SCOEL/SUM/124 2007 2007; Elwood 2021). Air concentrations of H_2_S from 0.2 to 29.4 ppm (0.3–41 mg/m^3^), noticeably higher than these TWA and STEL, were detected in thermal springs located in Ardabil Province, in a structure with several indoor pools (Fazlzadeh et al. 2018). The thermal site of *Contursi Terme* is characterized by outdoor pools only, so lower concentrations of H_2_S in the air are expected. In fact, passive sampling allowed us to detect concentrations ranging from 0.11 to 1.90 mg/m^3^ (Fig. 3) and these values present no risks for human health. These results can be explained by the fact that the air concentration of H_2_S is strongly related to the thermal spring emissions, while that of SO_2_ is probably derived also by other emission pathways.

However, active sampling performed near the spring source revealed that concentrations were, in some cases, higher than the limits in the working environment for both H_2_S and SO_2_.

This study has some limitations. First, the investigation is limited to monitoring a single site that has specific microclimate conditions and structural elements that are different from those of other thermal natural springs. Second, this study ruled out some factors that can affect local employee health during work hours in a thermal spring such as dietary habits, job description, lifestyle and smoking. Furthermore, numerous workers’ psychological, physical and health conditions were not included in the data collection. However, in this study, we focused on assessing the air quality, in terms of H_2_S and SO_2_ concentrations, to which workers at this spa complex are daily exposed. We believe that this approach is the first stage in safeguarding the health of the workers and to which should be given more consideration. To our knowledge, only one other example of H_2_S concentration monitoring in a spa area can be found in the literature. Undoubtedly, the above limitations must be taken into account in a quantitative assessment of the health effects on workers as a result of exposure to these pollutants.

## Conclusion

The chemical identification of pollutants in a particular environment, such as a thermal spring, and control of their concentration are essential to suggest new and improved procedures of safety and guidelines for professional activities. Although the water of thermal structures provides beneficial effects on human health, air monitoring, performed near the spring source, showed that concentrations of sulphurous compounds (both H_2_S and SO_2_) are in some cases higher than the limits in the working environment. Therefore, further investigations and regulations are needed to estimate the occupational risk and ensure workers’ safety in these particular working places.

## Supplementary Information

Below is the link to the electronic supplementary material.

Supplementary file1 (DOCX 30 KB)

## Data Availability

Not applicable.
