# Supplementary material for: Sulphurous air pollutants and exposure events of workers in thermal-mineral springs: a case study of Contursi Terme (Salerno, Italy)
Source: Environ Sci Pollut Res Int. 2022 Aug 9;30(2):3112–20. doi: 10.1007/s11356-022-22432-y (PMC9892073; doi:10.1007/s11356-022-22432-y)
Supplement: Supplementary file 1 — Supplementary file1 (DOCX 30 KB) [file 11356_2022_22432_MOESM1_ESM.docx]

**Supporting information**

**Sulphurous air pollutants and exposure events of workers in thermal-mineral springs: a case study of *Contursi Terme* (Salerno, Italy)**

Concetta Pironti^1^, Maria Ricciardi^1^, Oriana Motta^1*^, Marta Venier^2^, Antonio Faggiano^3^, Raffaele Cucciniello^3^, Antonio Proto^3^

^1^Department of Medicine Surgery and Dentistry, University of Salerno, via S. Allende, 84081 Baronissi (SA), Italy;

^2^O’Neill School of Public and Environmental Affairs, Indiana University, Bloomington, IN, United States

^3^Department of Chemistry and Biology, University of Salerno, via Giovanni Paolo II 132, 84084 Fisciano (SA), Italy.

^*^omotta@unisa.it

**Summary**

[1. Description of samples 2](#_Toc98397684)

[**Table S1**. Description of collected samples in Contursi Terme with exposure time, weather conditions and number of samples (N). 2](#_Toc98397685)

[2. Air concentrations by passive sampling 5](#_Toc98397686)

[**Table S2**. Air concentration values of SO_2_ and H_2_S (mean of three values) obtained by passive sampling in Contursi Terme, with the standard deviation (σ). 5](#_Toc98397687)

[3. Statistical analyses 6](#_Toc98397688)

[**Table S3**. ANOVA results for differences in concentration of SO_2_ between sampling periods. 6](#_Toc98397689)

[**Table S4**. ANOVA results for differences in concentration of SO_2_ between environments (outdoor-indoor). 6](#_Toc98397690)

[**Table S5**. ANOVA results for differences in concentration of H_2_S between sampling periods. 6](#_Toc98397691)

[**Table S6**. ANOVA results for differences in concentration of H_2_S between environments(outdoor-indoor). 7](#_Toc98397692)

# 1. Description of samples

## **Table S1**. Description of collected samples in Contursi Terme with exposure time, weather conditions and number of samples (N).

| Sample | Description | Sampling date | Exposure time (h) | Weather conditions | N |
| --- | --- | --- | --- | --- | --- |
| 1 | SO_2_ active sampling | 21 January 2015 | 1/3 | Rainy | 3 |
| 2 | SO_2_ active sampling | 30 January 2015 | 1/3 | Sunny and windy | 3 |
| 3 | SO_2_ active sampling | 11 February 2015 | 1/3 | Sunny and windy | 3 |
| 4 | SO_2_ active sampling | 18 February 2015 | 1/3 | Sunny and windy | 3 |
| 5 | SO_2_ active sampling | 13 March 2015 | 1/3 | Sunny and windy | 3 |
| 6 | SO_2_ active sampling | 24 March 2015 | 1/3 | Sunny and windy | 3 |
| 7 | SO_2_ active sampling | 1 April 2015 | 1/3 | Sunny and windy | 3 |
| 8 | SO_2_ active sampling | 10 April 2015 | 1/3 | Sunny and windy | 3 |
| 9 | H_2_S active sampling | 21 January 2015 | 1/3 | Rainy | 3 |
| 10 | H_2_S active sampling | 30 January 2015 | 1/2 | Sunny and windy | 3 |
| 11 | H_2_S active sampling | 11 February 2015 | 1/2 | Sunny and windy | 3 |
| 12 | H_2_S active sampling | 18 February 2015 | 1/2 | Sunny and windy | 3 |
| 13 | H_2_S active sampling | 13 March 2015 | 1/2 | Sunny and windy | 3 |
| 14 | H_2_S active sampling | 24 March 2015 | 1/2 | Sunny and windy | 3 |
| 15 | SO_2_ passive sampling indoor | 21 January 2015 -  30 January 2015 | 216 | Rainy | 3 |
| 16 | SO_2_ passive sampling outdoor | 21 January 2015 -  30 January 2015 | 216 | Rainy | 3 |
| 17 | SO_2_ passive sampling indoor | 30 January 2015 -  11 February 2015 | 288 | Sunny and windy | 3 |
| 18 | SO_2_ passive sampling outdoor | 30 January 2015 -  11 February 2015 | 288 | Sunny and windy | 3 |
| 19 | SO_2_ passive sampling indoor | 11 February 2015 - 18 February 2015 | 168 | Sunny and windy | 3 |
| 20 | SO_2_ passive sampling outdoor | 11 February 2015 - 18 February 2015 | 168 | Sunny and windy | 3 |
| 21 | SO_2_ passive sampling indoor | 18 February 2015 - 13 March 2015 | 336 | Sunny and windy | 3 |
| 22 | SO_2_ passive sampling outdoor | 18 February 2015 - 13 March 2015 | 336 | Sunny and windy | 3 |
| 23 | SO_2_ passive sampling indoor | 13 March 2015 -  24 March 2015 | 264 | Sunny and windy | 3 |
| 24 | SO_2_ passive sampling outdoor | 13 March 2015 -  24 March 2015 | 264 | Sunny and windy | 3 |
| 25 | SO_2_ passive sampling indoor | 24 March 2015 -  1 April 2015 | 192 | Sunny and windy | 3 |
| 26 | SO_2_ passive sampling outdoor | 24 March 2015 -  1 April 2015 | 192 | Sunny and windy | 3 |
| 27 | SO_2_ passive sampling indoor | 1 April 2015 -  10 April 2015 | 216 | Sunny and windy | 3 |
| 28 | SO_2_ passive sampling outdoor | 1 April 2015 -  10 April 2015 | 216 | Sunny and windy | 3 |
| 29 | SO_2_ passive sampling indoor | 10 April 2015 -  21 April 2015 | 264 | Sunny and windy | 3 |
| 30 | SO_2_ passive sampling outdoor | 10 April 2015 -  21 April 2015 | 264 | Sunny and windy | 3 |
| 31 | H_2_S passive sampling indoor | 21 January 2015 -  30 January 2015 | 216 | Rainy | 3 |
| 32 | H_2_S passive sampling outdoor | 21 January 2015 -  30 January 2015 | 216 | Rainy | 3 |
| 33 | H_2_S passive sampling indoor | 30 January 2015 -  11 February 2015 | 288 | Sunny and windy | 3 |
| 34 | H_2_S passive sampling outdoor | 30 January 2015 -  11 February 2015 | 288 | Sunny and windy | 3 |
| 35 | H_2_S passive sampling indoor | 11 February 2015 - 18 February 2015 | 168 | Sunny and windy | 3 |
| 36 | H_2_S passive sampling outdoor | 11 February 2015 - 18 February 2015 | 168 | Sunny and windy | 3 |
| 37 | H_2_S passive sampling indoor | 18 February 2015 - 13 March 2015 | 336 | Sunny and windy | 3 |
| 38 | H_2_S passive sampling outdoor | 18 February 2015 - 13 March 2015 | 336 | Sunny and windy | 3 |
| 39 | H_2_S passive sampling indoor | 13 March 2015 -  24 March 2015 | 264 | Sunny and windy | 3 |
| 40 | H_2_S passive sampling outdoor | 13 March 2015 -  24 March 2015 | 264 | Sunny and windy | 3 |
| 41 | H_2_S passive sampling indoor | 24 March 2015 -  1 April 2015 | 192 | Sunny and windy | 3 |
| 42 | H_2_S passive sampling outdoor | 24 March 2015 -  1 April 2015 | 192 | Sunny and windy | 3 |
| 43 | H_2_S passive sampling indoor | 1 April 2015 -  10 April 2015 | 216 | Sunny and windy | 3 |
| 44 | H_2_S passive sampling outdoor | 1 April 2015 -  10 April 2015 | 216 | Sunny and windy | 3 |
| 45 | H_2_S passive sampling indoor | 10 April 2015 -  21 April 2015 | 264 | Sunny and windy | 3 |
| 46 | H_2_S passive sampling outdoor | 10 April 2015 -  21 April 2015 | 264 | Sunny and windy | 3 |

# 2. Air concentrations by passive sampling

## **Table S2**. Air concentration values of SO_2_ and H_2_S (mean of three values) obtained by passive sampling in Contursi Terme, with the standard deviation (σ).

| **Sample** | **Analyte/ environment** | **Air concentration (mg/m^3^)** | **σ** |
| --- | --- | --- | --- |
| 15 | SO_2_ /indoor | 0.81 | 0.02 |
| 16 | SO_2_ /outdoor | 0.61 | 0.02 |
| 17 | SO_2_ /indoor | 0.91 | 0.02 |
| 18 | SO_2_ /outdoor | 0.70 | 0.04 |
| 19 | SO_2_ /indoor | 0.51 | 0.03 |
| 20 | SO_2_ /outdoor | 0.66 | 0.03 |
| 21 | SO_2_ /indoor | 0.49 | 0.03 |
| 22 | SO_2_ /outdoor | 0.61 | 0.03 |
| 23 | SO_2_ /indoor | 0.16 | 0.02 |
| 24 | SO_2_ /outdoor | 0.11 | 0.02 |
| 25 | SO_2_ /indoor | 0.41 | 0.03 |
| 26 | SO_2_ /outdoor | 0.33 | 0.04 |
| 27 | SO_2_ /indoor | 0.26 | 0.05 |
| 28 | SO_2_ /outdoor | 0.33 | 0.04 |
| 29 | SO_2_ /indoor | 0.26 | 0.04 |
| 30 | SO_2_ /outdoor | 0.19 | 0.02 |
| 31 | H_2_S /indoor | 0.21 | 0.03 |
| 32 | H_2_S /outdoor | 1.60 | 0.02 |
| 33 | H_2_S /indoor | 0.22 | 0.03 |
| 34 | H_2_S /outdoor | 0.65 | 0.03 |
| 35 | H_2_S /indoor | 0.18 | 0.02 |
| 36 | H_2_S /outdoor | 0.57 | 0.02 |
| 37 | H_2_S /indoor | 0.12 | 0.02 |
| 38 | H_2_S /outdoor | 0.40 | 0.03 |
| 39 | H_2_S /indoor | 0.11 | 0.03 |
| 40 | H_2_S /outdoor | 1.20 | 0.03 |
| 41 | H_2_S /indoor | 0.56 | 0.03 |
| 42 | H_2_S /outdoor | 0.47 | 0.03 |
| 43 | H_2_S /indoor | 0.21 | 0.04 |
| 44 | H_2_S /outdoor | 0.94 | 0.03 |
| 45 | H_2_S /indoor | 0.19 | 0.03 |
| 46 | H_2_S /outdoor | 1.90 | 0.03 |

# 3. Statistical analyses

Statistical analysis, one-way ANOVA (analysis of variance), was performed using R studio software

(version 4.1.1). In particular, we evaluated the statistical differences between the indoor and outdoor concentrations of the considered pollutants obtained by passive sampling and the statistical differences between the concentrations recorded in different sampling periods. The null hypotheses for the ANOVA analysis were that there are no differences between indoor and outdoor concentration values detected for the same pollutant during the same sampling period and there are no differences between concentration recorded in different sampling period. Hence, the independent variables were the “type of environment” (indoor and outdoor) and the “sampling period” (January-February and March-April), whereas the dependent variable was the air concentration of the considered pollutants. The significance level was α=0.05

## **Table S3**. ANOVA results for differences in concentration of SO_2_ between sampling periods.

|  | **Df** | **Sum Sq** | **Mean Sq** | **F value** | **Pr>F** |
| --- | --- | --- | --- | --- | --- |
| Sampling period | 3 | 0.7290 | 0.24301 | 21.64 | 3.94e-05 *** |
| Residuals | 12 | 0.1348 | 0.01123 |  |  |

## **Table S4**. ANOVA results for differences in concentration of SO_2_ between environments (outdoor-indoor).

|  | **Df** | **Sum Sq** | **Mean Sq** | **F value** | **Pr>F** |
| --- | --- | --- | --- | --- | --- |
| Sampling period | 1 | 0.0042 | 0.00422 | 0.069 | 0.797 |
| Residuals | 14 | 0.8595 | 0.06140 |  |  |

## **Table S5**. ANOVA results for differences in concentration of H_2_S between sampling periods.

|  | **Df** | **Sum Sq** | **Mean Sq** | **F value** | **Pr>F** |
| --- | --- | --- | --- | --- | --- |
| Sampling period | 3 | 0.737 | 0.2455 | 0.785 | 0.525 |
| Residuals | 12 | 3.754 | 0.3129 |  |  |

## **Table S6**. ANOVA results for differences in concentration of H_2_S between environments(outdoor-indoor).

|  | **Df** | **Sum Sq** | **Mean Sq** | **F value** | **Pr>F** |
| --- | --- | --- | --- | --- | --- |
| Sampling period | 1 | 2.198 | 2.1978 | 13.42 | 0.00256** |
| Residuals | 14 | 2.293 | 0.1638 |  |  |
